# Metabolic dysfunction and early‐onset colorectal cancer – how macrophages build the bridge

**DOI:** 10.1002/cam4.3315

**Published:** 2020-07-23

**Authors:** Katharina M. Scheurlen, Adrian T. Billeter, Stephen J. O'Brien, Susan Galandiuk

**Affiliations:** ^1^ Price Institute of Surgical Research Department of Surgery University of Louisville Louisville KY USA; ^2^ Department of General, Visceral, and Transplantation Surgery University Hospital Heidelberg Heidelberg Baden‐Wuerttemberg Germany

**Keywords:** adiposity, colonic neoplasms, colorectal neoplasms, obesity, rectal neoplasms, tumor‐associated macrophages

## Abstract

**Background:**

The incidence of colorectal cancer (CRC) among patients <50 years of age has increased dramatically over the last decades. At the same time, the growing proportion of obese children and adolescents and the increasing proportion of young and obese patients with CRC suggests an association between metabolic dysfunction and carcinogenesis. Tumor‐associated macrophages (TAMs) are able to orchestrate tumor promoting and suppressing mechanisms in CRC. The aim of this review was to discuss the different roles of TAMs in CRC and their phenotype‐specific metabolic pathways to identify potential new targets for CRC treatment.

**Methods:**

A literature search was performed using PubMed, Cochrane and Embase to identify studies on TAMs and their metabolism in CRC. The following search terms were used in various combinations: (obesity OR adiposity OR obese) AND (macrophage OR polarization OR macrophage metabolism) AND ((colon cancer*) OR (colon carcinoma) OR (colonic tumor*) OR (colonic neoplasm[MeSH]) OR (rectal cancer*) OR (rectal carcinoma) OR (rectal tumor*) OR (rectal neoplasm[MeSH]) OR (colorectal cancer*) OR (colorectal carcinoma) OR (colorectal tumor*) OR (colorectal neoplasm[MeSH])). Studies including data on the phenotype and metabolism of TAMs in CRC were analyzed.

**Results:**

Evidence for the prognostic utility of macrophage markers in CRC is currently evolving, with a particular role of stage‐dependent cellular metabolism profiles of TAMs. Itaconate is one of the metabolites produced by proinflammatory subtypes of TAMs and it is known to have tumor promoting effects. Metabolic pathways that are involved in macrophage activation and reprogramming play a role in a chronic inflammatory setting, consequently affecting the onset and development of CRC.

**Conclusions:**

Tumor‐promoting metabolites, such as itaconate, are directly regulating these mechanisms, thereby triggering carcinogenesis. Metabolic reprogramming in TAMs can build a bridge between metabolic dysfunction and the onset and progression of CRC through inflammatory pathways, particularly in younger patients with early‐onset CRC.

## Graphical abstract

The incidence of colorectal cancer (CRC) among patients <50 years of age has increased dramatically over the last decades. At the same time, the growing proportion of obese children and adolescents and the increasing proportion of young and obese patients with CRC suggests an association between metabolic dysfunction and carcinogenesis. Metabolic reprogramming in TAMs can build a bridge between metabolic dysfunction and the onset and progression of CRC through inflammatory pathways, particularly in younger patients with early‐onset CRC.

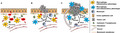

## 1 INTRODUCTION

Colorectal cancer (CRC) is one of the most common causes of cancer‐related death in the USA and its incidence among young adults has increased over the last several decades.(1, 2, 3, 4) Simultaneously, the proportion of overweight and obese children and adolescents is rising, suggesting an association between metabolic dysfunction and carcinogenic mechanisms.(4, 5, 6, 7) A direct association between obesity and early‐onset CRC was found among young women as part of the The Nurses' Health Study II.(4) Comparing obese and non‐obese individuals among different cancer types, CRC was the only type of cancer that showed an increase in young adults with obesity undergoing CRC resections.(8)

Most cases of CRC are either sporadic in etiology, based on a somatic mutation involving Adenomatous polyposis coli gene (*APC*) function, or less often hereditary, as in hereditary nonpolyposis colorectal cancer (HNPCC) or familial adenomatous polyposis (FAP).(9, 10) HNPCC is the most common cause of hereditary CRC.(11) While the proportion of other hereditary cancer syndromes in patients with early‐onset CRC is widely unknown, HNPCC represents 4%‐13.5% of cancers in patients with early‐onset CRC.(11) Only a small percentage of CRC is actually associated with inflammatory bowel disease such as ulcerative colitis or Crohn's disease.(12, 13) Compared to 0.4% of patients with a late onset of CRC, young patients with CRC showed an increased prevalence of inflammatory bowel disease in 3% of cases.(14) There is, however, strong evidence that anti‐inflammatory medication can reduce the risk of CRC, even in the absence of an underlying inflammatory bowel disease.(15, 16) Furthermore, low‐dose aspirin therapy is associated with a reduced risk of advanced stage disease, which might indicate that anti‐inflammatory medication also suppresses CRC progression.(17) This suggests that inflammatory processes may have a greater role in the onset and development of CRC than previously assumed.

As essential components of the immune inflammatory response, macrophages are able to orchestrate inflammatory mechanisms and therefore tumorigenesis. The tumor microenvironment (TME) is a dynamic environment surrounding the tumor and a critical part of these regulatory processes. Tumor cells, immune cells and the blood and lymphatic vascular networks interact with stromal cells and their extracellular matrix, coordinating cancer establishment, tumor growth and metastasis.(18) Current evidence shows that tumor‐associated macrophages (TAMs) play a central role in the dynamic processes within the TME, contributing to tumor promoting effects as well as contributing to tumor suppressing mechanisms in CRC.(18) Various functions require dynamic switching between different TAM phenotypes. These phenotypes depend upon specific metabolic pathways within TAMs, which provide a source for functional metabolites and facilitate phenotype‐specific inflammatory or anti‐inflammatory activities during cancer development.(19, 20) The impact of cell interactions within the TME on a patient's outcome, including risk of CRC recurrence or effectiveness of cytoreductive chemotherapy, radiotherapy, or immunotherapy, is poorly understood.

The aim of this review was to highlight current publications and trendsetting approaches regarding metabolic dysfunction and TAMs in CRC, and to discuss their phenotype‐specific metabolic pathways in order to identify pathogenetic mechanisms as potential new targets for early‐onset CRC treatment.

## 2 SYSTEMIC METABOLIC DYSFUNCTION AND ITS LINK TO COLORECTAL CANCER

The incidence of overweight and obesity in the general population is increasing worldwide with a remarkable rising trend in children and young adolescents.(21) Simultaneously, an increase in CRC rates among younger adults <50 years of age is reported.(1, 2, 4) Regular screening for CRC is recommended by the U.S. Preventive Services Task Force (USPSTF) beginning at age 50.(22) Due to the large‐scale screening programs in these patients >50 years of age with removal of precancerous polyps during colonoscopy and earlier detection of CRC, the incidence and mortality of CRC has declined.(23, 24) The increasing number of colon cancer cases among younger adults has, however, led to the recommendation of an earlier start of regular screening by the American Cancer Society (ACS) in 2018, beginning at age 45.(25) The parallel increase in the incidence of obesity in the young and early‐onset CRC indicates that obesity‐related inflammatory mechanisms may play a greater role in the development of CRC than assumed.

Overweight and obesity are defined as an abnormal fat accumulation with a respective Body Mass Index (BMI) of ≥25 and ≥30 kg/m^2^ by the World Health Organization (WHO).(21) Obesity is an established risk factor for type 2 diabetes mellitus and its associated complications.(26) The underlying metabolic dysfunction is due to chronic systemic inflammation that can lead to insulin‐resistance.(27) During the last three decades, epidemiological data and several cohort and case‐control studies have shown an association between obesity and CRC.(28, 29, 30) The simultaneously rising incidence of obesity and CRC in patients younger than 50 years of age indicates a particular contributing role of metabolic dysfunction in the development early‐onset CRC.(4) Liu et al prospectively analyzed a patient cohort of more than 85 000 women aged 25 to 42 years, that were part of the The Nurses' Health Study II.(4) An association between obesity and early‐onset CRC was found among this patient collective. The recent analysis of Hussan et al showed an increasing trend in CRC among young patients with obesity, that could not be demonstrated in other types of gastrointestinal cancer.(8) Focusing on the age of diagnosis in patients with CRC, an increased cancer risk was shown after a diagnosis of type 2 diabetes, especially in men younger than 55 years.(31)

A recent study on the molecular characteristics of early‐onset CRC showed that inflammatory mechanisms, such as deregulated redox homeostasis as one of the hallmarks of CRC in young patients, play a distinct role.(32) The major pathways that are involved in these mechanisms are altered Nuclear factor erythroid 2‐related factor 2 (NRF)‐mediated oxidative stress response, glutathione metabolism, and the chemokine (C‐X‐C motif) ligand 12 ‐ C‐X‐C motif chemokine receptor 4 (CXCL12‐CXCR4) signaling axis.(32) These findings suggest that metabolic dysfunction and obesity represent an important contributing factor in CRC development in young patients.

A chronic inflammatory environment is caused by the proinflammatory endocrine activity of adipose tissue, affecting energy homeostasis and glucose metabolism.(33) Inflammatory macrophages can accumulate within adipose tissue in obese patients and trigger inflammation, which leads to systemic metabolic dysfunction, including insulin resistance.(34) The presence of macrophages is a hallmark of proinflammatory adipose tissue. They form crown‐like structures in subcutaneous and visceral fat deposits.(34) Furthermore, adipose tissue‐derived inflammatory mediators have been shown to induce macrophage polarization toward a proinflammatory phenotype in an in vitro model.(35) In other obesity‐related comorbidities, such as nonalcoholic fatty liver disease (NFLD) or steatohepatitis (NASH), inflamed adipose tissue has been associated with activation of liver macrophages as a determinant for liver fibrosis.(36) Proinflammatory macrophage polarization in tissue macrophages can provide a link between the proinflammatory systemic state in obesity and a chronic inflammatory environment in colon tissue, which in turn can trigger carcinogenic mechanisms in colon epithelium through inflammatory stress.

Itaconate is a macrophage‐specific metabolite, which is produced in proinflammatory macrophages, and which is known to have tumor promoting effects.(37) TAMs in tumor‐bearing mice as well as monocytes isolated from patients with ovarian cancer showed increased itaconate production.(37)

Identifying the role of macrophage metabolism and itaconate in a chronic inflammatory state due to metabolic dysfunction and obesity, could lead to innovative approaches to screening diagnosis and treatment of CRC.

### 2.1 Chronic Inflammation in colorectal cancer

Chronic inflammation is closely linked to two systems of the human body that have major roles for survival: the immune system with the ability to fight infection and the metabolic system that can provide stored energy during a period of low nutrition.(27) Immunity and metabolism are therefore in a continuous state of interplay through inflammatory pathways. Both systems share several mediators, including hormones, cytokines, transcription factors, signaling proteins, and lipids. A chronic inflammatory state functions as a stressor and promotes tissue damage that can lead to neoplasia. Once a genetic mutation leads to oncogene activation, inflammation will contribute to cell proliferation, tumor establishment, growth, and metastasis. CRC is a cancer type known to be closely associated with chronic inflammation. Even though less than 2% of CRC is colitis‐associated, sporadic CRC shows similar mutations in genes and signaling pathways, such as the Wnt/β‐catenin pathway, K‐Ras or B‐Raf activation, adenomatous polyposis coli (*APC*) inactivation, transforming growth factor(TGF)‐β, P53, and the DNA mismatch repair (MMR) proteins.(38, 39, 40, 41) The pathogenesis of both CRC types differs in the histological sequence that is followed during development of neoplasia and the initiation of cancer formation.

Numerous clinical and epidemiological studies have shown that the use of aspirin or nonsteroidal anti‐inflammatory drugs (NSAIDs) is associated with a reduced risk of CRC or recurrent adenomatous polyps as well as decreased CRC mortality.(17, 42, 43, 44, 45, 46) Furthermore, low‐dose aspirin therapy seems to slow progression of a tumor that is already established. A recent cohort study of more than 300 000 patients in the United Kingdom demonstrated that new use of low dose aspirin was associated with a reduced risk of advanced stage CRC (Duke's B‐D) at diagnosis.(17) In 2015, the USPSTF started recommending low‐dose aspirin for chemoprevention of CRC in patients with increased cardiovascular risk aged 50‐59 years.(47)

Independent of its pathogenesis, CRC is infiltrated by immune cells such as macrophages, neutrophils or lymphocytes, that induce and maintain cancer‐related inflammation.(48)

In colon adenomas, the precursor lesions of sporadic CRC, TAMs with low major histocompatibility complex class 2 (MHC II) expression were observed, and the density of these macrophages correlates with tumor progression.(49) This suggests that mechanisms within the TME lead to macrophage polarization toward an anti‐inflammatory phenotype during the development of cancer. Furthermore, high‐grade adenomas have been shown to consist of a higher fraction of anti‐inflammatory macrophages than low‐grade adenomas.(50) This leads to the conclusion that macrophages of an anti‐inflammatory type seem to have a role in malignant transformation of colorectal adenomas toward CRC.

The link between immunity and cancer through inflammation was observed as early as the 19th century by the German pathologist Rudolf Virchow, when he described white blood cells as part of the tumor mass. In 1986, the American pathologist Harold Dvorak investigated angiogenesis within tumors, considered these mechanisms similar to those in wounds and depicted tumors as ‘wounds that do not heal’.(51) Inflammatory tissue injury causes chemotactic signaling that attracts immune cells to repair damage, and TAMs are the major cell type orchestrating the pathways within the TME, to either promote or suppress tumor development in CRC.(52) These opposing functions of TAMs are characterized by a respective dominating metabolic pathway of the macrophage that can be affected by extracellular signals within the tumor environment. This polarization into different functional subsets can be affected by proinflammatory cytokines,(53, 54) leading to the conclusion, that there is a direct connection between metabolism, inflammation and macrophage differentiation affecting tumor behavior.

Itaconate is a metabolite within inflammatory macrophages, and a regulator of cellular metabolism as well. It regulates glycolysis and leads to succinate accumulation through inhibition of succinate dehydrogenase.(55) This can lead to decreased production of reactive oxygen species (ROS) and altered activation of numerous transcription factors, such as nuclear factor kappa‐light‐chain‐enhancer of activated B cells (NF‐κB), hypoxia‐inducible factor 1α (HIF1α), signal transducer and activator of transcription 3 (STAT3), and activator protein 1 (AP‐1).(37, 55) NRF2 is a superordinate regulator of these anti‐inflammatory functions, that is affected by itaconate.(55)

In anti‐inflammatory macrophages, itaconate can further boost anti‐inflammatory functions.(55) Since anti‐inflammatory macrophages play a role in tumor progression in CRC, this suggests that itaconate affects CRC growth.

## 3 TUMOR‐ASSOCIATED MACROPHAGES IN COLORECTAL CANCER—BASICS FROM THE BENCH

The ability of macrophages to adapt to various environments and to provide a wide variety of functions in tissue is due to dynamic adjustments of their cellular metabolism. These metabolic pathways can be affected by the particular TME inducing the metabolic reprogramming, which in turn leads to different cell phenotypes.

### 3.1 Cellular metabolism and different phenotypes of tumor‐associated macrophages

**Figure 1:**
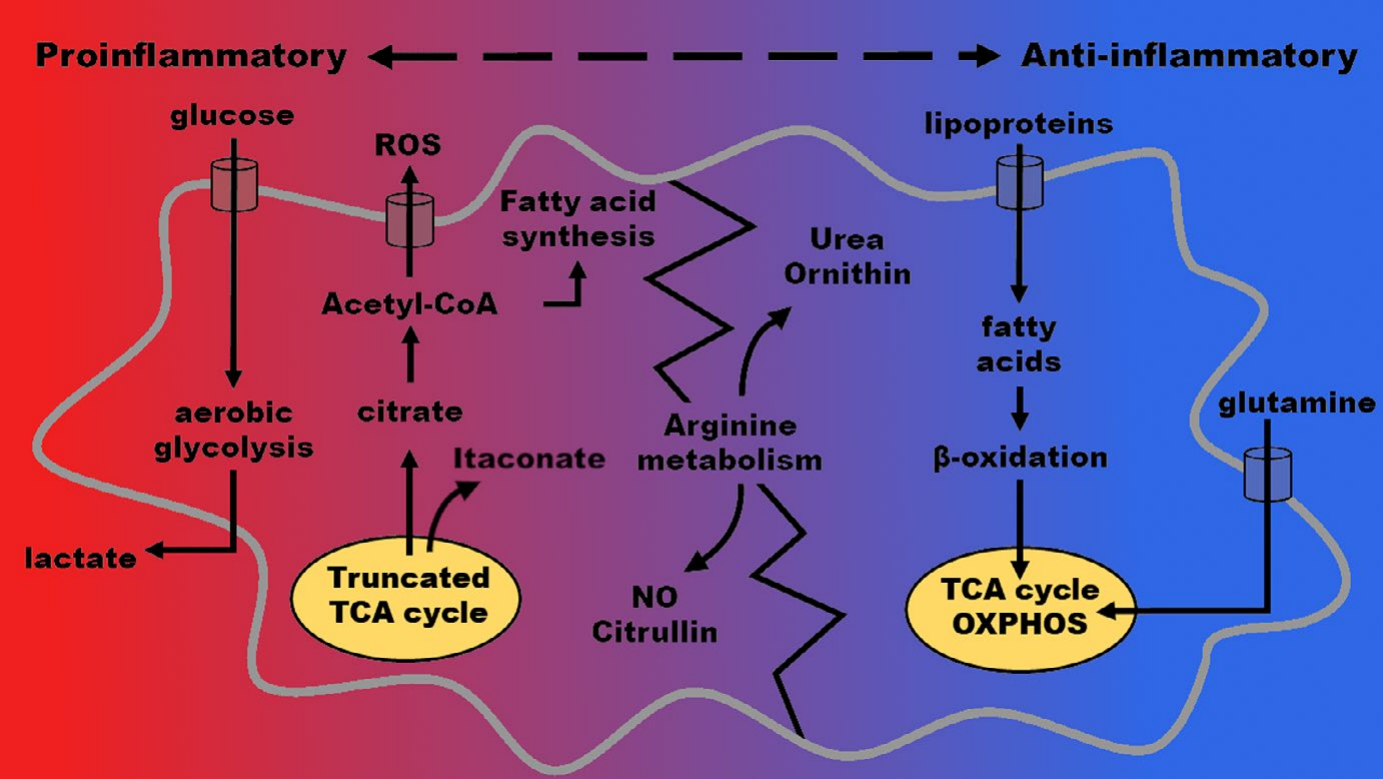
Metabolic pathways in proinflammatory and anti‐inflammatory phenotypes of tumor‐associated macrophages. Simplified model showing the dominating metabolic pathways in both extremes of phenotypes in tumor‐associated macrophages. As macrophages can switch between proinflammatory and anti‐inflammatory phenotypes continuously by changing their cellular metabolism, metabolic pathways can overlap between both types. Proinflammatory macrophages focus on aerobic glycolysis, truncated tricarboxylic acid cycle (TCA cycle) and fatty acid synthesis for energy homeostasis of the cell. Anti‐inflammatory macrophages use the TCA cycle, oxidative phosphorylation and β‐oxidation as their major energy sources. ROS: reactive oxygen species; TCA cycle: tricarboxylic acid cycle

Metabolic reprogramming can occur as a result of different stimuli on TAMs, for example, mediators secreted by cancer cells, signals from cells within the tumor microenvironment, self‐secretion or indirect stimuli such as hypoxia. Although switching between phenotypes is a continuous transition with intermediate types present, two main macrophage phenotypes have been described: an M1‐subtype with primarily inflammatory functions and an M2‐subtype, with predominantly anti‐inflammatory and immunosuppressive activity (Figure 1). This simplified classification is an attempt to distinguish between subsets of macrophages that have a primarily—but not exclusively—inflammatory or anti‐inflammatory function. The ‘waterfall model’ illustrates specific characteristics of TAMs during their development from a monocyte to an anti‐inflammatory macrophage subtype.(56) During this process, monocytes that initially present markers, such as C‐C chemokine receptor type 2 (CCR2) and lymphocyte antigen 6 complex (Ly6C), undergo functional and therefore phenotypical changes, losing Ly6C, and gaining MHC II expression.(56) This demonstrates the continuous transition of monocytes and macrophages with overlapping cell surface markers during all stages of development.

Depending upon their phenotype, macrophages prefer specific metabolic pathways for their energy homeostasis. The characteristic metabolic profiles of inflammatory and anti‐inflammatory macrophages lead to distinct phenotypes with respect to cellular metabolism, which can be studied instead of targeting cell surface markers (Table 1). While aerobic glycolysis is the main pathway in proinflammatory macrophages receiving M1 stimuli, anti‐inflammatory M2 macrophages are characterized by slower rates of aerobic glycolysis and primarily fatty acid oxidation.(57, 58) The classically activated inflammatory M1 macrophages show induction of glycolysis through the AKT/mTOR/HIF pathway.(37) Aerobic glycolysis is an inefficient pathway with a high rate of glucose consumption, but it is essential for rapid energy production and biosynthesis. M1 macrophages utilize this pathway for host‐defense against pathogens, including the production of ROS to kill bacteria or tumor cells. A slower rate of aerobic glycolysis within M2 macrophages is necessary for the production of cytokines.(58) In contrast to M1 macrophages, the M2 subset macrophages show increased oxidative phosphorylation (OXPHOS).(37) As shown in hepatocellular carcinoma, cancer cells can promote glycolysis in M2 macrophages through soluble mediators, increasing the gene expression of the glycolytic enzyme *PFKFB3*.(59) Therefore, glycolysis plays a role in both macrophage phenotypes, but the respective energy production focuses on different glycolysis‐associated pathways.

**Table 1:** Human macrophage characteristics depending on their metabolic phenotype (inflammatory versus anti‐inflammatory)

Phenotype	Proinflammatory (M1‐like subtype)	Anti‐inflammatory (M2‐like subtype)
Cell surface markers	CD11c, CD16, CD80, CD86, MHC II	CD163, CD206, CD209
Factors inducing differentiation	IFN‐γ, TNF, LPS, ATP	IL‐4, IL‐10, IL‐13, TGF‐β
Metabolic pathways	Aerobic glycolysis, truncated TCA cycle (Itaconate production), fatty acid synthesis	β‐oxidation, oxidative TCA cycle
Secreted factors	IL‐1β, IL‐6, IL‐8, IL‐12, IL‐23, IL‐27, TNF‐α, CXCL1, CXCL9, CXCL10, CXCL11, CCL2, CCL5, RNI, ROI, COX2	IL‐10, IL‐13, IL‐1RA, TGF‐β, CCL17, CCL18, CCL22, CCL24, Arg1, COX1, VEGF, PDGF

Note

The listed cell surface markers, factors and metabolic pathways are not exclusively present in only one of these macrophage phenotypes. Since macrophages can switch between phenotypes showing fluent transitions, these characteristics might overlap. However, the characteristics that are shown in this table are more likely to be present in the respective phenotype.

Abbreviations: Arg1: arginase 1; ATP: *adenosine* thiotriphosphate; CCL: CC‐chemokine ligand; CD: cluster of differentiation; COX: cyclooxygenase; CXCL: chemokine (C‐X‐C motif) ligand; IFN: interferone; IL: interleukin; LPS: lipopolysaccharides; MHC II: major histocompatibility complex class 2; PDGF: platelet‐derived growth factor; RNI: reactive nitrogen intermediates; ROI: reactive oxygen intermediates; TCA cycle: tricarboxylic acid cycle; TGF: transforming growth factor; TNF: tumor‐necrosis factor; VEGF: vascular endothelial growth factor.

### 3.2 The dual role of tumor‐associated macrophages in colorectal cancer

**Figure 2:**
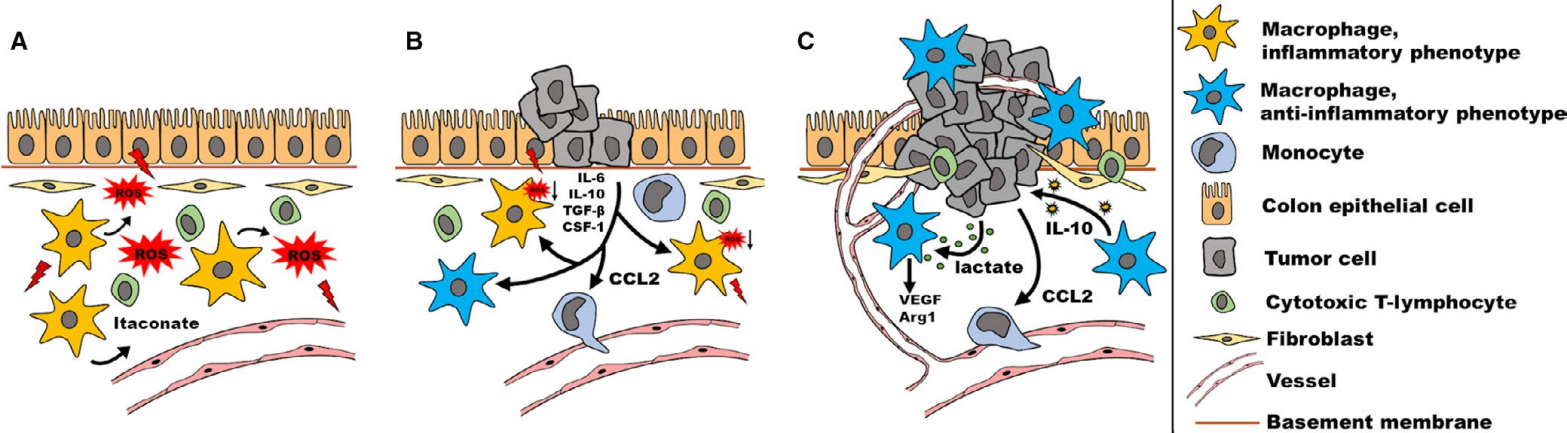
Tumor‐associated macrophages (TAMs) in chronic inflammation and colorectal cancer (CRC) development. (A) Tissue‐resident macrophages with a proinflammatory phenotype might be able to trigger the onset of CRC in the presence of a mutagenic activation of oncogenes in colon epithelial cells due to inflammatory stress and itaconate production. (B) During early cancer development colon cancer cells produce chemokines (CCL2) to attract bone marrow‐derived monocytes and induce macrophage differentiation releasing cytokines and growth factors such as IL‐6, IL‐10, TGF‐β, and M‐CSF (CSF‐1). (C) Colon cancer cells release mediators such as lactate to induce TAM polarization into an anti‐inflammatory phenotype. Reprogrammed macrophages show an increased expression of vascular endothelial growth factor (VEGF) and Arginase 1 (Arg 1), promoting angiogenesis and tumor growth. Furthermore, anti‐inflammatory TAMs promote tumor development by inducing IL‐10 production in colon cancer cells. ROS: reactive oxygen species; IL: interleukin; TGF: transforming growth factor; M‐CSF: Macrophage colony‐stimulating factor

In contrast to other solid human cancers, TAMs in colorectal cancer seem to have the ability to both support and suppress tumor growth. Tumor‐promoting mechanisms are known to result from an interplay between cancer cells, the tumor microenvironment and TAMs. It is hypothesized, that tumor initiation is fostered by mutagenic mechanisms from a chronic inflammatory environment in the subepithelial stroma.(60) Proinflammatory M1 macrophages that produce reactive oxygen and nitrogen species, are able to potentiate this effect, triggering oncogenic mutations in the adjacent epithelial layer (Figure 2). Once neoplasia is initiated, the tumor recruits additional bone marrow‐derived monocytes from the bloodstream and stimulates myelopoiesis by releasing growth factors and chemotactic signals such as CC‐chemokine ligands 2 and 5 (CCL2, CCL5), vascular endothelial growth factor (VEGF) and transforming growth factor beta (TGF‐β).(60, 61, 62) In adipose tissue, a similar mechanism is described, where CCL2 expression leads to increased macrophage infiltration and inflammation, which in turn is associated with insulin resistance.(63) Macrophage colony stimulating factor‐1 (M‐CSF or CSF‐1) has been shown to be produced by colon cancer cells in order to attract and ‘re‐educate’ macrophages.(62) During the early stages of tumor development, neoplastic cells seem to first attract monocytes and ensure their maturation to macrophages within the TME. After their differentiation to TAMs, cancer cells take these macrophages hostage by manipulating their metabolism through multiple signaling pathways and use these TAMs to support further tumor growth and progression. Overexpression of the chemoattractant CCL2 has been associated with advanced tumor stages, metastatic disease and poor prognosis in CRC.(64, 65) Furthermore, CRC cells produce lactic acid as a by‐product of predominantly aerobic glycolysis.(66) Proliferating cancer cells switch their metabolism toward aerobic glycolysis, which is known as the ‘Warburg effect’. Irrespective of the availability of oxygen, they metabolize glucose to lactate, which is also secreted to induce VEGF and arginase 1 (*ARG1*) expression in TAMs.(66) VEGF expression in macrophages was shown to be upregulated by a pathway described in hypoxia, even under normoxic conditions.(66) This mechanism leads to macrophage recruitment and polarization toward the tumor promoting M2 macrophage phenotype and is therefore associated with metabolic reprogramming in TAMs. Another key mechanism for the alternative activation of tissue macrophages is the peroxisome proliferator activated receptor‐γ (PPARγ) pathway.(67) In animal studies, the disruption of this pathway also was associated with diet‐induced obesity, insulin resistance, and glucose intolerance.(67) PPARγ deficiency can also lead to increased itaconate production, which suggests that itaconate acts as an alternative regulator of M2‐like polarization.(55)

Furthermore, signal transducer and activator of transcription 3 (STAT3) activation leads to M2 polarization of macrophages.(68) This pathway can be induced by glucagon‐like peptide 1 (GLP‐1), a postprandially secreted hormone that improves insulin resistance.(68) TAMs also promote tumor development by inducing interleukin 10 (IL‐10) production in CRC cells through a STAT3 pathway(69, 70) and produce cytokines such as VEGF to induce tumor angiogenesis and tumor growth.(71)

### 3.3 Tumor‐associated macrophages as prognostic predictors in colorectal cancer

The density of recruited macrophages and their metabolic phenotype were found to be associated with different clinical outcomes in CRC patients. Despite the heterogeneity among study methods used to investigate the degree of TAM infiltration, a high TAM density within the primary tumor is associated with an improved prognosis in CRC patients.(72) In other solid tumors, such as gastric, urogenital and head and neck cancers, a high TAM density is accompanied by worse overall survival.(72) A higher degree of infiltrating macrophages in the invasive front of CRC, in particular those with an M1 phenotype, is associated with a better prognosis in a stage‐dependent manner.(73, 74) Furthermore, it is inversely correlated to lymph node and liver metastases.(75, 76) While M2 macrophages seem to be more prevalent in stage II CRC, M1 macrophages are predominant in less invasive T1 tumors.(77) This indicates that M1 macrophages are primarily responsible for tumor initiation because of inflammatory mechanisms increasing oncogenic potential. Further in the course of the tumor, cancer cells recruit additional bone marrow‐derived blood monocytes and reprogram their metabolism to induce M2‐polarization.(60)

Investigating the different functions and phenotypes of TAMs during tumor development, which in turn promote and suppress tumor growth, is the basis for developing new diagnostic and therapeutic targets, especially in early‐onset CRC. The specific role of itaconate, that can regulate macrophage polarization in tumors, is currently unknown in CRC. Table 2 provides an overview of current studies investigating TAM phenotypes and therefore indirectly TAM metabolism in CRC.

**Table 2:** Studies on colorectal cancer investigating cancer‐related mechanisms related to the phenotype and metabolism of tumor‐associated macrophages

Author	Year of publication	Study model	Aims & objectives	Results	Conclusions regarding TAM metabolism
Colegio et al [66]	2014	Murine/murine cell line	Identification of tumor signals that lead to functional polarization of macrophages Lactic acid levels in cell line media The effect of lactic acid on macrophage polarization towards an anti‐inflammatory M2‐like phenotype using syngeneic LLC tumors M2‐marker expression by macrophages induced by lactic acid	Tumor cells induce VEGF & Arginase1 expression in macrophages via HIF1α Comparison of intracellular metabolites of M1‐line & M2‐like macrophages revealed that lactate & pyruvate levels were most different Lactic acid induced *FIZZ1*, *MGL1* & *MGL2* in bone‐marrow derived macrophages	Tumor cell‐derived lactic acid had an important signaling role in macrophage polarization & therefore tumor growth Lactic acid induced expression of genes that are defined as markers of M2‐like anti‐inflammatory macrophages (VEGF, Arginase 1, *FIZZ1*, *MGL1* & *MGL2*)
Deng et al [90]	2010	Murine	Effect of blocking *STAT3*‐mediated signaling in macrophages in a transgenic mouse line Tumor development in inflamed colon & cecum in *STAT3* knockout mice & the effect of microflora on these processes	20% of *STAT3* knockout mice showed pronounced colitis at 8 weeks of age; the rate further increased in older mice. Macrophage density increased in the colon of these mice The inflamed colon developed visible polyps with associated carcinoma A higher density of intestinal microflora was found in the stool of *STAT3* knockout mice compared to controls	*STAT3* knockout promotes tissue inflammation in the colon of this transgenic mouse line, suggesting a role in macrophage polarization towards an M1‐like proinflammatory subtype Abnormal immunity in the bowel mucosa might induce changes in the microflora
Edin et al [73]	2012	Human tissue	Identification of macrophage phenotypes in CRC in relation to prognosis in CRC in general & in subgroups defined by microsatellite instability (MSI) screening status & CpG island methylator phenotype (CIMP)	The amount of iNOS positive and CD163 positive cells both correlated inversely with tumor stage A higher amount of iNOS positive cells associated with better prognosis, independent of MSI & CIMP status No significant survival associations found in groups of CRC with different iNOS/CD163 ratios	A higher density of iNOS positive cells (M1 subtype, proinflammatory) is accompanied by a higher amount of CD163 positive cells (M2‐like subtype, anti‐inflammatory) & correlated with better prognosis in a stage dependent manner
Feng et al [86]	2019	Human tissue	Macrophage density & proportion of CD206 positive macrophages as prognostic/predictive biomarkers in stage II colon cancer	A high CD206/CD68 ratio was associated with poor disease‐free survival & poor overall survival CD206/CD68 ratio had a better prognostic efficacy than density of macrophages (CD68 positive cells), or of CD206 positive macrophages or other clinicopathologic high‐risk factors Disease‐free survival & overall survival were improved in patients with a high CD206/CD68 ratio receiving adjuvant chemotherapy, but not in patients with a low CD206/CD68 ratio receiving adjuvant chemotherapy	In stage II colon cancer, a higher proportion of CD206 positive macrophages (M2‐like subtype, anti‐inflammatory) in relation to the overall density of macrophages (CD206/CD68 ratio) is associated with poor disease‐free & overall survival rates. A higher proportion of anti‐inflammatory M2‐like macrophages (higher CD206/CD68 ratio) is associated with beneficial effects on survival in stage II colon cancer patients that receive adjuvant chemotherapy
Herbeuval et al [69]	2004	Human cell lines	Interactions between macrophages & tumor cells including IL‐6, IL‐10 & *STAT3* activation	Media of cultured macrophages can stimulate IL‐10 production in several human colon adenocarcinoma cell lines through a mechanism involving IL‐6 Recombinant IL‐6 (but not recombinant IL‐10), TNFα and IFNα stimulated IL‐10 secretion in colon cancer cell lines IL‐10 gene regulation was mediated by *STAT3*. This mechanism was regulated by IL‐6	Mediators released by macrophages (with proinflammatory effects, M1‐like subtype) induced *STAT3*‐mediated IL‐10 production by colon cancer cells that can lead to M2‐polarization of macrophages
Koelzer et al [85]	2016	Human tissue	Intra‐tumoral & stromal macrophage density in CRC Direct cell contact between cancer cells (tumor buds) & macrophages Predominant macrophage phenotype in CRC	A higher density of intraepithelial macrophages correlated with less tumor budding A higher density of stromal macrophages correlated with larger tumor diameter & less lymph node metastasis Frequent contact between tumor buds & macrophages was present in tumors with: higher grade, lymph node metastasis, mismatch repair deficiency & BRAF mutation as well as in patients without adjuvant therapy 40% of macrophages (CD68 positive) were also CD163 positive, 60% were iNOS positive High counts of CD163 positive macrophages were associated with lower tumor grade, less lymph node metastasis, less advanced T‐stage, absence of lymphatic invasion, KRAS wild type genotype & a non‐significant survival benefit	Macrophage phenotypes, classified by cell surface markers, show an association with survival. A high CD163 positive macrophage count (M2‐like subtype, anti‐inflammatory) was associated with a non‐significant survival benefit. Proinflammatory iNOS positive macrophages (M1‐like subtype) showed no association with survival
Malesci et al [87]	2017	Human tissue/human cell lines	Macrophage density at the invasive front of the primary tumors & metastatic lymph nodes Prognostic/predictive value of macrophages & neutrophils & interactions with 5‐fluorouracil adjuvant therapy	High macrophage densities in primary tumors & lymph nodes were associated with a lower risk of tumor recurrence after resection as well as with better disease‐free survival in 5‐fluorouracil treated patients Patients with stage III CRC & high macrophage density in tumors (particularly metastatic lymph nodes) show significantly better 5‐year‐disease‐free survival than patients with low macrophage density Cancer cell death was not increased by 5‐fluorouracil exposure after coculturing with unpolarized macrophages Coculturing cancer cells with M1‐like macrophages nearly doubled the cell death rate. This was even further increased by exposure to 5‐fluorouracil	Proinflammatory macrophages (M1‐like subtype) & 5‐fluorouracil showed a synergistic effect on cancer cell death
Nandi et al [91]	2016	Murine	Effects of *CCR6* knockout on growth of a syngeneic transplanted colon cancer in mice Macrophage density in syngeneic transplanted colon cancer in *CCR6* deficient mice Effect of CCL20 on macrophage accumulation in vivo Effect of macrophage accumulation on tumor growth in *CCR6* deficient mice Correlations between CCR6 expression with that of the macrophage marker CD163 & with CCL2, IL‐1α, IL‐6 & TNFα	Macrophage density was lower & tumor growth was delayed in *CCR6* deficient mice compared to wild type mice Macrophage accumulation was greater in response to CCL20 than to CCL2 Macrophage depletion led to reduced tumor growth Higher macrophage density in wild type mice accompanied by increased expression of CCL2, IL‐1, IL6, but not TNFα, compared to *CCR6* deficient mice Expression of *CCR6* correlated with CD163, CCL2, IL‐1α & TNFα	Macrophages accumulating in response to CCL20 and *CCR6* interaction secrete proinflammatory factors (M1‐like subtype) Expression of *CCR6* correlates with the expression of cell surface marker CD163 (M2‐like subtype, anti‐inflammatory)
Oosterling et al [92]	2005	Murine	Comparison of mRNA expression profiles, tumor load & survival between animals with macrophage‐depleted tumors & controls Identification of macrophage phenotypes within tumor tissue	Macrophage‐depleted tumors showed higher differentiation & reduced inflammatory tumor infiltrates Higher tumor load in the peritoneal cavity & liver observed in macrophage‐depleted animals Augmented tumor development in macrophage‐depleted rats correlated with decreased survival of these animals, supporting the significance of macrophage tumoricidal effector functions. General macrophage density higher throughout control tumors compared to macrophage‐depleted tumors CD163 positive macrophages confined to the tumor periphery in control tumors & no CD163 positive cells found in macrophage‐depleted tumors	Macrophage depletion leads to higher tumor load, reduced inflammatory tumor infiltrates & to loss of the anti‐inflammatory macrophage population present in control tumors (M2‐like subtype)
Pinto et al [77]	2019	Human tissue	Identification of macrophage phenotypes in different stages of CRC	The amount of macrophages, especially CD163 positive macrophages, was high in stage II CRC The amount of CD80 positive macrophages was higher in less invasive T1 tumors & is associated with lower risk of cancer recurrence Higher macrophage density and lower CD80/CD163 ratio were associated with impaired overall survival	CD163 positive macrophages (M2‐like subtype, anti‐inflammatory) predominated in higher tumor stages and were associated with worse overall survival CD80 positive macrophages (M1‐like, proinflammatory) were associated with lower tumor stage & lower risk of recurrence
Umemura et al [93]	2008	Murine/murine cell line	Macrophage phenotype identification in murine colon adenocarcinoma	Tumor‐infiltrating monocytes/macrophages had CCR2 positive & CX3CR1 positive inflammatory monocyte characteristics Tumor‐infiltrating monocytes/macrophages were shown to produce TGF‐β1, which led to upregulation of CD206 expression	Tumor‐infiltrating monocytes/macrophages cannot be classified into M1 and M2 categories since they bear overlapping characteristics CD206 expression (M2‐like subtype, anti‐inflammatory) in tumor‐infiltrating monocytes/macrophages is regulated by an autocrine mechanism using TGF‐β1
Zhou et al [75]	2010	Human tissue	Association between CD68 hotspots (small areas with infiltration of CD68 positive cells above the average level of CD68 positive cell infiltration) and other clinicopathologic parameters, potential of hepatic metastasis, & 5‐year survival Macrophage phenotypes within tumor tissue	CD68 hotspots were prognostic for survival & were associated with the potential of hepatic metastasis & the interval between colon resection & the occurrence of hepatic metastasis Patients with stage IIIB cancer & higher macrophage density in the invasive front of the tumor had a higher 5‐year survival rate after resection Staining for identification of macrophage phenotypes showed a large proportion of HLA‐DR, IL‐10 & IL‐12 positive macrophages, a smaller proportion of TGF‐β1 positive macrophages & absence of IL‐12 positive macrophages.	Macrophage density at the invasive front of a tumor is associated with lower potential of hepatic metastasis & worse overall survival in colon cancer Macrophages within tumors predominantly expressed HLA‐DR (M1‐like subtype, proinflammatory) & IL‐10 (M2‐like subtype, anti‐inflammatory). Fewer cells were TGF‐β1 positive (M2‐like subtype, anti‐inflammatory). Clear conclusions on an M1‐like or M2‐like overall subtype by analyzing cell surface markers could not be drawn

Note

Abbreviations: BRAF: B‐Raf proto‐oncogene; CCL: CC‐chemokine ligand; CCR: C‐C Motif Chemokine Receptor; CCR6: C‐C Motif Chemokine Receptor 6; CD: cluster of differentiation; CRC: colorectal cancer; CXCL: chemokine (C‐X‐C motif) ligand; FIZZ1: found in inflammatory zone 1; HIF: hypoxia‐inducible factor; HLA‐DR: human leukocyte antigen‐DR; IL: interleukin; iNOS: inducible nitric oxide synthase; KRAS: Kirsten rat sarcoma viral oncogene homolog gene; LLC: Lewis Lung Carcinoma; MGL: macrophage galactose‐type lectin‐1; MHC II: major histocompatibility complex II; RELM α; STAT3: Signal transducer and activator of transcription 3; TGF‐β1: Transforming growth factor beta 1; TNF: tumor‐necrosis factor; VEGF: vascular endothelial growth factor.

## 4 PERSPECTIVES FOR TARGETING TUMOR‐ASSOCIATED MACROPHAGES IN CLINICAL PRACTICE

### 4.1 Tumor‐associated macrophages as diagnostic markers

TAMs have the potential to be used as diagnostic and prognostic markers in CRC and possibly as therapeutic targets. Previous studies have shown that circulating TAMs and the chemokines that they produce could serve as markers in cancer diagnosis.(78, 79, 80) Current research has focused on the identification of circulating TAMs in blood samples by profiling their cell surface markers in different types of cancer as a basis for developing a noninvasive screening tool. Relevant markers are cluster of differentiation (CD) 14, CD163, CD68 or hypoxia‐inducible factor 2α (HIF‐2α).(78, 79, 80) A combination of analyzes of cell surface markers, cytokines secreted by TAMs and soluble factors produced by other cells within the TME could be useful to determine specific cell expression profiles in CRC. Serum levels of neutrophil elastase within the TME have been shown to play a potential role as a diagnostic biomarker in CRC.(81) While serum matrix metalloproteinase‐9 (MMP‐9) was not considered to be an appropriate screening parameter for CRC,(82) tissue inhibitor of metalloproteinase‐1 (TIMP‐1) seems to have a potential diagnostic value.(83) Targeting related factors that are expressed by TAMs or neighboring cells within the TME and circulatory markers may further contribute to the overall diagnostic capacity.

### 4.2 Tumor‐associated macrophages as prognostic markers

Evidence for prognostic utility of markers in CRC, with a particular role of TAM phenotypes in different tumor stages, is currently evolving. The findings with respect to the association between specific TAM phenotypes and prognosis are inconclusive, suggesting a different role of TAMs during tumor progression. This could also be caused by the fact, that not only the total cell count of either M1 or M2 macrophages seems to be relevant for tumor progression, but also the distribution of these cells within the tumor environment.(73) A low density of TAMs in general, as investigated by CD68+ cell infiltration in tumor tissue, was associated with worse outcome in patients with different stages of CRC.(84) A high proportion of CD163+ macrophages was associated with lower tumor grade and less lymph node metastasis.(85) Other studies report advanced tumor stages and worse prognosis positively correlating with high TAM density.(77) An investigation of the prognostic effect of TAMs in patients with CRC undergoing postoperative chemotherapy recently revealed, that the CD206/CD68 ratio of TAMs can predict high risk of recurrence in patients with stage II colon cancer.(86) As adjuvant chemotherapy is not routinely recommended in these patients, identifying those patients with poor prognosis is leading to targeted and more accurate administration of chemotherapy. The presence of a high density of TAMs in primary tumor tissue and metastatic lymph nodes of stage III CRC can identify patients that benefit from 5‐fluorouracil.(87) In‐vitro results indicating synergistic effects of TAMs and fluoropyrimidines have, however, yet to be proven in an in‐vivo setting.(87)

Since different TAM phenotypes are associated with tumor behavior, the metabolic reprogramming of TAMs to an ‘antitumor’ phenotype is a major aim of ongoing research. In TAMs, the NF‐κB pathway is the main pathway for polarization into an antitumor phenotype. This pathway is affected by Toll‐like receptors, Dectin‐1 receptors and SIGN‐related 1 receptors.(88, 89) Activation of these receptors causes an adaptive immune response enhancing phagocytosis and the release of inflammatory cytokines, such as tumor‐necrosis factor α (TNFα), IL‐2, IL‐10, and IL‐12.(89) The yeast‐derived polysaccharide β‐glucan can act on these membrane receptors, thereby inducing macrophage polarization into a proinflammatory anticancer phenotype.(89) Apart from NF‐κB, other transcription factors can also be regulated to induce M1‐like polarization or to inhibit M2 polarization in macrophages, such as interferon‐regulatory factor (IRF), STAT protein, HIFα and several microRNAs.(54)

Pathways that are known to be involved in macrophage activation and reprogramming in the acute immune response could also play a role in a chronic inflammatory setting, consequently affecting the onset and development of CRC. Identifying inflammatory mediators in obesity that support the polarization of tumor‐promoting macrophages could not only help identify patients at high risk of CRC due to metabolic dysfunction, but also serve as a basis for targeting these mediators in patients with obesity or type 2 diabetes mellitus. The effects of obesity and its associated inflammatory stressors on macrophage polarization,

TAM metabolism and therefore tumor behavior in patients with CRC, need further elucidation.

## 5 CONCLUSIONS

Tumor‐promoting inflammation is one of the hallmarks of cancer and TAMs are able to orchestrate these mechanisms based on their cellular metabolism. Interactions between TAMs, tumor cells and other components within the TME regulate cancer establishment, tumor growth and metastasis. CRC is closely related to chronic tissue inflammation. Metabolic dysfunction in patients with obesity has the potential to induce reprogramming in TAMs through inflammatory mechanisms. The macrophage metabolite itaconate is produced during TAM polarization and it is known to have tumor promoting effects. Investigating the role of itaconate and other metabolites in TAMs can elucidate processes specific for the onset and progression of CRC on the basis of inflammatory pathways, particularly in early‐onset CRC. There is a potential to detect new diagnostic and prognostic targets for the improvement of neoadjuvant and/or adjuvant therapies in CRC.

## CONFLICTS OF INTEREST

K.M.S., A.T.B., S.J.O., and S.G. declare no potential conflicts of interest.

## AUTHOR CONTRIBUTION

K.M.S. and S.J.O. performed the literature search (data curation), K.M.S. and A.T.B. identified relevant studies (formal analysis), K.M.S. and S.G. wrote and edited the paper and all authors were involved in final draft changes. K.M.S. and S.G. developed the study and were in charge of overall direction and planning (project administration).

## Data Availability

The authors confirm that the data supporting the findings of this study are available within the article.
